# Low Hydrophobic Mismatch Scores Calculated for HLA-A/B/DR/DQ Loci Improve Kidney Allograft Survival

**DOI:** 10.3389/fimmu.2020.580752

**Published:** 2020-10-29

**Authors:** Dulat Bekbolsynov, Beata Mierzejewska, Jadwiga Borucka, Robert S. Liwski, Anna L. Greenshields, Joshua Breidenbach, Bradley Gehring, Shravan Leonard-Murali, Sadik A. Khuder, Michael Rees, Robert C. Green, Stanislaw M. Stepkowski

**Affiliations:** ^1^ Department of Medical Microbiology and Immunology, University of Toledo, Toledo, OH, United States; ^2^ Parexel International, Warsaw, Poland; ^3^ Department of Pathology, Dalhousie University, Halifax, NS, Canada; ^4^ Department of Surgery, Henry Ford Hospital, Detroit, MI, United States; ^5^ Department of Medicine and Public Health, University of Toledo, Toledo, OH, United States; ^6^ Department of Urology, University of Toledo College of Medicine, Toledo, OH, United States; ^7^ The Alliance for Paired Donation, Maumee, OH, United States; ^8^ Department of Computer Science, Bowling Green State University, Bowling Green, OH, United States

**Keywords:** ****kidney transplantation, kidney allocation, transplant survival, human leukocyte antigen (HLA)****, HLA mismatch, immunogenicity

## Abstract

We evaluated the impact of human leukocyte antigen (HLA) disparity (immunogenicity; IM) on long-term kidney allograft survival. The IM was quantified based on physicochemical properties of the polymorphic linear donor/recipient HLA amino acids (the Cambridge algorithm) as a hydrophobic, electrostatic, amino acid mismatch scores (HMS\AMS\EMS) or eplet mismatch (EpMM) load. High-resolution HLA-A/B/DRB1/DQB1 types were imputed to calculate HMS for primary/re-transplant recipients of deceased donor transplants. The multiple Cox regression showed the association of HMS with graft survival and other confounders. The HMS integer 0–10 scale showed the most survival benefit between HMS 0 and 3. The Kaplan–Meier analysis showed that: the HMS=0 group had 18.1-year median graft survival, a 5-year benefit over HMS>0 group; HMS ≤ 3.0 had 16.7-year graft survival, a 3.8-year better than HMS>3.0 group; and, HMS ≤ 7.8 had 14.3-year grafts survival, a 1.8-year improvement over HMS>7.8 group. Stratification based on EMS, AMS or EpMM produced similar results. Additionally, the importance of HLA-DR with/without -DQ IM for graft survival was shown. In our simulation of 1,000 random donor/recipient pairs, 75% with HMS>3.0 were re-matched into HMS ≤ 3.0 and the remaining 25% into HMS≥7.8: after re-matching, the 13.5 years graft survival would increase to 16.3 years. This approach matches donors to recipients with low/medium IM donors thus preventing transplants with high IM donors.

## Introduction

The impact of the human leukocyte antigen (HLA)-A/B/DR mismatch (MM) on kidney allograft survival has been a subject of extensive research (1–4). While some researchers argue that the role of HLA matching is exaggerated (5), the consensus is that HLA compatibility between a donor and a recipient (HLA immunogenicity) affects long-term kidney outcomes (6). Fully matched (0-HLA-A/B/DR MM) transplants consistently demonstrated the best kidney allograft survival, and fully mismatched (6-HLA-A/B/DR MM) transplant had the worst outcomes, whereas the remaining 1-, 2-, 3-, 4-, and 5-HLA MM groups had incrementally distributed survival between 0- and 6-HLA MM cohorts (1, 2). The graft half-life for kidney transplant from deceased donors was reported as 9.3 years in 2005 and 9.9 years in 2010 (7). Kaplan–Meier and Cox models showed that HLA disparity and multiple other factors may affect kidney allograft survivals (8).

In the U.S., most of ABO-compatible kidney transplants are performed without HLA matching, with the exception for few special programs (5, 9, 10). Between 2012 and 2016, only 16.4% of patients received “well-matched” deceased donor kidney transplants at 0-, 1- or 2-HLA MM. The remaining 83.6% of kidney transplants had 3-, 4-, 5-, and 6-HLA MM and therefore should be considered as poorly matched (11). Existing government programs prioritize 0-HLA MM kidney transplants for sensitized patients (12). In particular, the 0-MM benefits patients with HLA-A/-B/-DR matched donors who have a panel reactive antibody (PRA)>20%. From December 2014, the kidney allocation system (KAS) targets patients with a PRA≥98% and patients waiting ≥10 years on dialysis. The remaining procured kidneys are allocated according to UNOS policies that rarely grant points for HLA matches. Indeed, it is logistically impossible to match HLA for every transplant, and thus less than 10% of patients receive 0-HLA MM kidney transplants every year (13).

Not all HLA mismatches are equal, however. The idea of weakly immunogenic HLA mismatches has been used for 30 years in the Acceptable Mismatch program, benefiting over 1,000 very highly sensitized patients (14). Increasing availability of high-resolution HLA typing encourages analysis of molecular disparities in HLA for quantifying HLA immunogenicity beyond antigenic mismatches. One of resulting concepts was HLAMatchmaker for finding immunogenic HLA eplets (15). Eplets represent polymorphic HLA amino acids recognized by T and B cells (15). Alternatively, the Cambridge algorithm is based on the physicochemical properties of polymorphic HLA amino acid residues (16, 17). This algorithm quantifies HLA disparities based on its hydrophobic (HMS), electrostatic (EMS) and amino acid (AMS) properties. These physicochemical-based scores correlated with eplet MM load numbers, as well as the quality of alloantibody response and kidney allograft rejection (18, 19), or graft-*versus*-host disease (GVHD)-free survival after bone marrow transplantation (20). Conceptually, an association was recently shown between donor three-dimensional EMS value and the generation of specific antibody response (21). While other algorithms for HLA IM quantitation exist, the practical and clinically-relevant IM calculation needs to be developed (22).

With decreasing costs and shortening testing times, the 4-digit high-resolution HLA typing is an upcoming revolution in transplantation. Recently published rapid testing techniques may soon make it feasible for deceased donor transplants (23). Implementing 4-digit HLA typing has a potential to better avoid unacceptable antigens for sensitized patients. Herein, we explored an additional power of 4-digit HLA typing to provide a benefit of weakly immunogenic transplants and thus improve the long-term graft survival for all patients.

We examined the potential of 4-digit HLAs for finding weakly immunogenic donors. As opposed to the traditional seven integer 0–6-HLA mismatch (MM) system, a continuous physicochemical IM scale based on 4-digit HLA types in HLA-A/B/DRB1/DQB1 loci converts antigen mismatch categories into numerical values on a continuous scale. We propose that a continuous IM scale offers greater freedom in finding weakly immunogenic transplants, ultimately allowing improvement of kidney allograft survival. Using the Scientific Registry of Transplant Recipients (SRTR) data, we calculated the HMS, EMS, and AMS by the Cambridge algorithm as well as eplet MM (EpMM) by the HLAMatchmaker for class I (HLA-A/B) and class II (HLA-DRB1/DQB1) HLAs to correlate their values with the long-term kidney allograft survival. We used the Cox regression model to confirm an association between HMS, EMS, AMS, EpMM scores, graft survival, and multiple covariates. Furthermore, we created a simulated allocation model to check whether low/medium immunogenic ABO-compatible donors may be identified for all recipients in comparison to a small fraction of low IM donors identified by an inflexible 0–6 HLA MM system.

## Materials and Methods

### Patient Population

**Figure 1:**
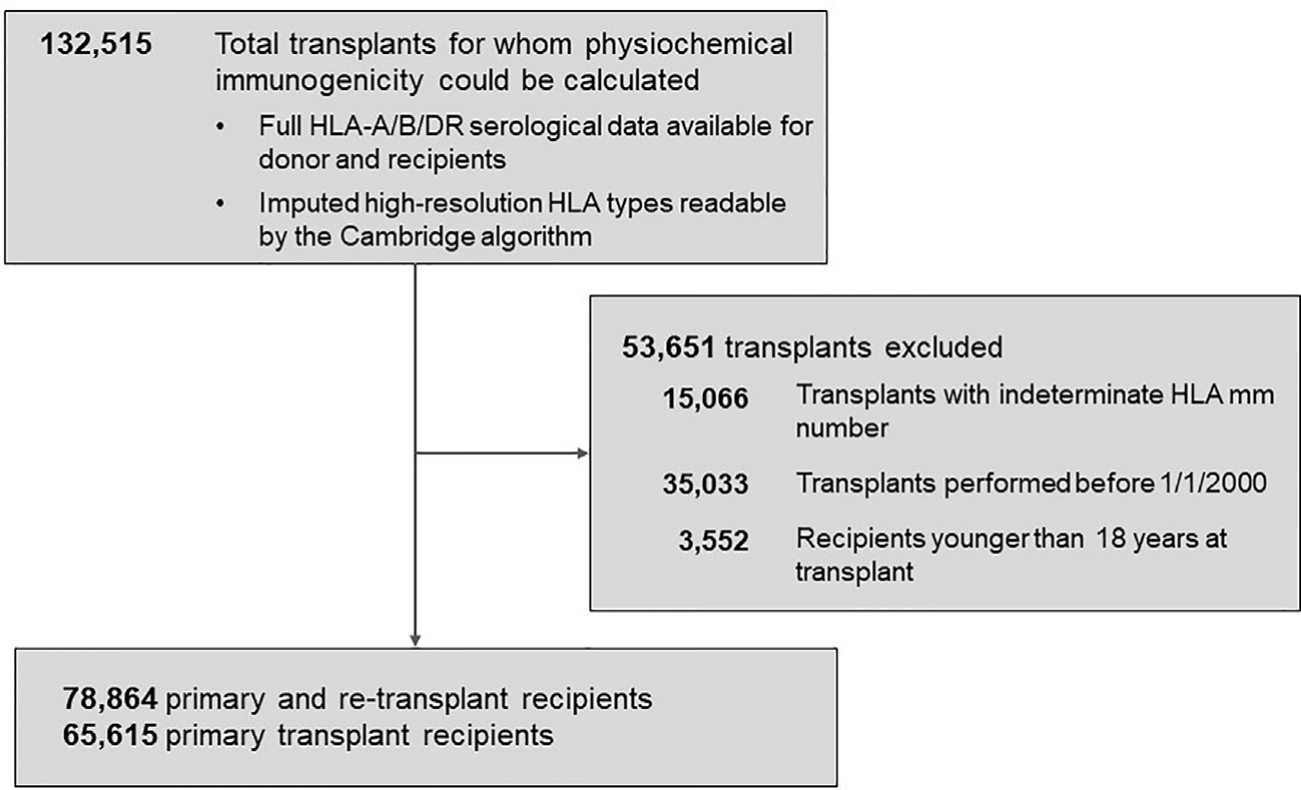
Background characteristics of patient groups represented in the study. The total population of 78,864 patients included 65,615 recipients with primary kidney transplants and 13,249 with re-transplants. Further details are presented in *Materials and Methods*.

The Scientific Registry of Transplant Recipients (SRTR) database was the source of transplant records between 01/01/2000 and 09/04/2016 (**Figure 1**). Out of all 132,515 recipients of deceased donors, high-resolution HLAs were imputed for 78,864 donor/recipient pairs based on available race and HLA-A/B/DR 2-digit antigens; this cohort included 29,852 pairs with HLA-A/B/DRB1/DQB1 types available. The 78,864-cohort had 65,615 patients with primary and 13,249 patients with repeat transplants.

**Disclaimer**. The SRTR data include all donors, wait-list candidates, and transplant recipients submitted by the members of the Organ Procurement and Transplantation Network (OPTN). The Health Resources and Services Administration (HRSA) is the U.S. Department of Health and Human Services providing an oversight to the activities of the OPTN and SRTR contractors.

### Imputation of High-Resolution HLA Types and Calculation of Immunogenicity

The HaploStats algorithm of the NMDP bioinformatics group was used to impute high-resolution HLA-A/B/DRB1/DQB1 to assign the possible corresponding high-resolution HLA types (24, 25). The frequency of HLA haplotypes was calculated based on the distribution of race in the 2011 NMDP full database (26): the most frequent high-resolution haplotype was assigned to the individual. The high likelihood of imputed of HLA alleles was recently demonstrated on a 5,000 cohort of patients in the U.K, namely 91% for HLA-A, 88% for -B, 67% for -DR, and 95% for -DQ, producing an average of 85% (27).

The physicochemical IM for each donor-recipient pair was calculated using the Cambridge algorithm, which assigns a score to each amino acid mismatch between donor and recipient HLA (16, 17). HLA-A/B/DRB1 or HLA-A/B/DR/DQB1 was used to quantitate the hydrophobic (HMS), electrostatic (EMS), and amino acid (AMS) mismatch scores: IM scores for each transplant were calculated as averages for class I (HLA-A/B) and class II (DR ± DQ) loci. That way, 50% of the IM score was contributed by DR alone or DR/DQ combination, as was suggested before (28).

In addition, eplet mismatch (EpMM) scores were calculated in version 3.1 of HLAMatchmaker. Sums of antibody-verified eplet loads in HLA-DRB1 and HLA-A/B loci were used as EpMM values in the sub-cohort of 47,241 patients.

To verify the accuracy of high-resolution HLA types imputation, a cohort of transplant candidates from Queen Elizabeth II Health Sciences Centre, Halifax, Canada (n = 1,095) typed to 2-field, P-group resolution at HLA-A/B/C/DRB1/DQB1 loci using a combination of sequence based typing and extended region SSO methods was used as a reference. Imputed and known high-resolution HLA types and immunogenicity values calculated based on them were then statistically evaluated.

### Statistical Analyses

Graft loss for death-censored graft survival analyses was defined as reported graft failure, resumption of dialysis, re-transplant, or listing for re-transplant. Deaths with functioning graft were censored. Graft half-lives or median survival (time points where 50% of transplants survived) was established using Kaplan–Meier estimates. When follow-up time was shorter than the graft half-life, a linear survival curve was projected starting from day 4,000 post-transplant.

Four immunogenicity cutoffs were applied to define immunological risk categories that were tested in Kaplan–Meier survival estimates (see **Table 3**). Within the HMS scale, the “perfect allelic match” was HMS=0, the “predictive threshold” was HMS=1.8 [established by maximally selected rank statistics method, (29)], the “weakly immunogenic” threshold was HMS=3.0 (equal to average HMS in patients with 0-/1-HLA MM) and the “median” threshold was HMS=7.8. Analogous thresholds were defined individually for EMS, AMS and EpMM scales. The same approach was used for HLA MM scale.

Multiple Cox regression models analyzed the association of graft loss risk with immunogenicity and other confounders (**Table 1**). All data analyses were performed in SAS 9.4 (SAS Institute Inc., Cary, NC) and R (R Foundation for Statistical Computing, Vienna, Austria) programs.

**Table 1:** Baseline characteristics of patients included in the study (n = 78,864).

Variable	n	%
Donor age		
Less than 18	8,399	10.7
18 to 34	23,256	29.5
35 to 49	24,073	30.5
50 to 64	20,278	25.7
65 and over	2,858	3.6
Recipient age		
18 to 34	9,275	11.8
35 to 49	22,053	27.9
50 to 64	33,348	42.3
65 and over	14,188	18.0
Donor gender		
Male	46,442	58.9
Female	32,422	41.1
Transplant era		
2000–2004	18,121	23.0
2005–2016	60,743	77.0
Induction		
Yes	61,523	78.0
No	16,587	21.0
Unknown	754	1.0
Maintenance immunosuppression*		
TAC/PRED	2,556	3.2
CsA/MMF/PRED	5,497	7.0
TAC/MMF/PRED	57,851	73.3
Other	316	11.9
Multiple regimens	9,359	0.4
Unknown	3,285	4.2
Recipient race		
White	36,435	46.2
African American	27,618	35.0
Asian	4,131	5.2
Hispanic/Latino	9,721	12.3
Other	959	1.3
Donor race		
White	62,376	79.1
African American	13,064	16.6
Asian	1,891	2.4
Hispanic/Latino	976	1.2
Other	557	0.7
Recipient education		
None, grade school or high school	37,073	47.0
Attended college, bachelors or post-college	32,207	40.8
Unknown	9,584	12.2
Recipient primary source of payment		
Private insurance	31,976	40.5
Public insurance	34,302	43.5
Other	12,370	15.7
Unknown	216	0.3
Cold ischemia time		
Less than 24 hours	58,092	73.7
24 hours or more	15,508	19.7
Unknown	5,264	6.6
Waiting time		
Less than 1 year	25,497	32.3
1 to 3 years	29,350	37.2
3 to 5 years	15,980	20.3
Over 5 years	8,037	10.2
Candidate BMI > 30		
Yes	27,745	35.2
No	29,510	62.8
Unknown	1,609	2.0
Candidate pre-transplant diabetes		
No	51,811	65.7
Type I	2,482	3.2
Type II	16,069	20.4
Other	8,329	0.4
Unknown	173	10.3
Pre-transplant dialysis		
No dialysis	15,219	19.3
Hemodialysis	45,586	57.8
Peritoneal Dialysis	8,345	10.6
Continuous Venous/Venous	739	0.9
Hemofiltration		
Unknown	8,975	11.4

*PRED indicates the use of steroids or steroid-free regimen.

### Building the Proportional Hazards Model

A semi-parametric multiple Cox proportional hazards model estimated the death-censored graft failure as a function of HLA immunogenicity and other confounders. Univariate regression models identified 16 confounders that were significantly associated with death-censored graft survival (**Table 1**), using a previously described approach (30). With the exception for pre-transplant dialysis and recipient education level, all confounders had less than 10% of missing values; records with missing values were excluded.

All selected variables were tested for the proportional hazard assumption using ASSESS statement of PHREG procedure in SAS. This statement simulated 1,000 possible components *vs.* follow-up time plots (31, 32). The Kolmogorov-type supremum test was performed to establish the deviation from the simulated plots of score process components and time, indicating a possible violation of proportionality assumption. Additional verification was performed using Schoenfeld residuals and log-negative log plots for each variable (not shown). Variables violating the proportional hazard assumption were included in the Cox model with time log transformation (33).

### Simulated Allocation Model

We have designed a simulated allocation algorithm to test the maximum number of donors that could be found for recipients within a designated HMS threshold. When the HMS was greater than the designated threshold, the pair was broken and simulation re-run with other broken pairs until the number of pairs under the designated HMS threshold stopped growing. Thus, a maximum number of low IM pairs within a given population was established. Four input populations (n =10, n = 100, n = 1,000, or n = 5,000) and five IM thresholds (HLA MM ≤ 1 or HMS = 0, HMS ≤ 3.0, HMS ≤ 5.5 or HMS ≤ 7.8) were tested. This procedure was repeated 10 times always with new pairs for all except 5,000 pairs, which was run only once as re-matching was prohibitively lengthy.

## Results

### Patient Demographics and 4-Digit HLA Imputation

A total of 78,864 kidney primary and re-transplants from the SRTR were included into cohort. Baseline characteristics of the cohort are presented in **Table 1**. There were 30,526 (38.7%) female recipients and 48,338 (61.3%) male recipients with mean age at transplant of 51.9 years; 13,553 patients died with a functioning kidney. Among donors, 32,422 (41.1%) were female 46,442 (58.9%) were male; their mean age was 38.4 years. Recipients were represented by mostly non-Hispanic whites (46.2%), while 35.0% were black. Recipients who had at least a high school education or less represented most of patients (47.0%), whereas 40.8% of patients attended college. Most patients (80.7%) were on hemodialysis prior transplantation.

To assure the accuracy of our analyses, the same imputation from 2-digit to 4-digit HLA-A/B/DRB1/DQB1 types was performed for the individuals typed at Queen Elizabeth II Health Sciences Centre with high resolution at HLA-A/B/DRB1/DQB1 loci (n = 1,095). The imputed high-resolution was 96% identical in HLA-A locus, 92% in -B locus, 73% in -DRB1 locus, and 85% in -DQB1 locus (**Table 2**). In addition, we used the above individuals in an experiment to test the impact of imputed HLAs ***vs.*** real HLAs on the accuracy of IM calculation in our analysis: 547 presumed donors and 547 presumed recipients were used to calculate HMS/EMS/AMS values. In paired t-test, there was no difference between HMS (p = 0.31), EMS (p = 0.36), and AMS (p = 0.37) values of imputed ***vs***. real 4-digit HLA types. Thus, the HLA imputation had little, if any, impact on this proof-of-concept results (**Table 2**).

**Table 2:** Identity between the known and imputed high-resolution types in the 1,095 patients from Haifax (upper part) and similarity between physicochemical immunogenicity levels calculated based on them (lower part).

Identity of imputed high-resolution HLA types to the laboratory results									
HLA Locus		A		B		DR		DQ	
**% of Identical typing**		96.1		92.7		73.8		85.3	
**Immunogenicity calculated based on real or imputed high-resolution HLA types**									
**Immunogenicity measure**		**HMS**		****	**EMS**		****	**AMS**	
		**Real**	**Imputed**		**Real**	**Imputed**		**Real**	**Imputed**
**Mean**		12.04	11.91		12.22	12.11		8.23	8.17
**Paired t-test p-value**		0.316			0.362			0.369	

### Association of HLA Mismatch Levels With HMS Immunogenicity Scores

**Figure 2:**
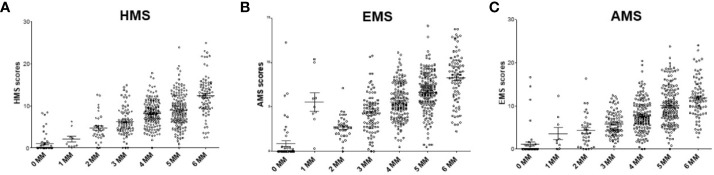
The distribution of physicochemical HMS, EMS, and AMS mismatch (scores across the antigenic 0–6-HLA MM groups. Each dot represents 200 donor–recipient pairs; Panels **(A)** (HMS), **(B)** (EMS) and **(C)** (AMS) has 78,864 recipients of primary or repeat kidney allografts. More details in *Materials and Methods*.

Currently, the HLA IM between a donor and a recipient is defined at 2-digit level as 0–6 HLA-A/B/DR MM. We investigated the association of kidney allograft survival where HLA IM was defined at 4-digit level by the Cambridge algorithm (16, 17). The HMS, EMS, and AMS MM scores strongly correlated based on intra-class correlation coefficient of 82.5% (HMS/EMS linear regression at R^2^ = 0.90), as previously reported (16). When HMS, EMS, and AMS values were plotted on the 0- to 6-HLA MM scale, the 1- and 2-HLA MM groups had the lowest whereas the 5- and 6-HLA MM groups had the highest average HMS values (**Figures 2A–C**). However, while the low IM scores were found predominantly among 0-, 1-, and 2-HLA MM pairs, they were also present in 3-, 4-, 5- and 6-HLA MM groups. The same was true for highly IM transplants, high HMS values were found in the 1- to 6-HLA MM groups. Similar HMS distribution patterns were in the primary and re-transplant cohorts (not shown).

### Multiple Cox Regression Analysis

A semi-parametric multiple Cox proportional hazard method estimated the association of HMS values with other confounders on the risk for graft failure. Selected confounders showed a significant association with the death-censored graft survival in univariate Cox regression models (**Supplemental Table 1**). Those variables were then tested for the proportional hazard assumption using ASSESS statement of PHREG procedure in SAS (31, 32), and variables violating the proportional hazard assumption were included with time log transformation (33). All HLA MM, and HMS values were significantly associated with graft loss. In conclusion, the HMS-measured IM had significant association with the death-censored graft survival in multiple regression models (**Supplemental Table 2**). In addition, immunosuppression, induction therapy, PRA levels, and cold ischemia time were associated with the risk of graft loss (p < 0.001; **Supplemental Table 2**).

### Low Immunogenicity Scores in HLA-A/B/DR Correlate With Better Kidney Allograft Survival

**Figure 3:**
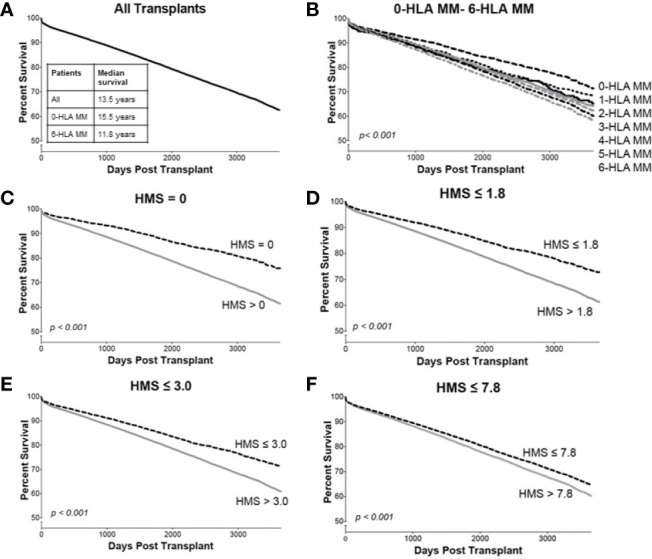
The Kaplan–Meier death-censored graft survival as a function of HLA immunogenicity in primary/re-transplant cohort (n = 78,865). Panel **(A)** presents the unadjusted primary/re-transplant cohort; Panel **(B)** presents patients stratified by 0–6-HLA mismatches; Panel **(C)** presents patients with HMS=0 *vs*. patients with HMS>0; Panel **(D)** presents patients with HMS ≤ 1.8 *vs*. patients with HMS>1.8; Panel **(E)** presents patients with HMS ≤ 3.0 *vs*. patients with HMS>3.0; and Panel **(F)** presents patients with HMS ≤ 7.8 *vs.* HMS>7.8. More details in *Materials and Methods*.

Overall, our 78,864 cohort had a 10-year kidney transplant survival of 62.3% with half-life of 13.5 years (**Figure 3A**). Traditional 0- to 6-HLA MM plots showed an incremental decrease in death-censored graft survival (**Figure 3B**): 0-MM had 71% 10-year survival with half-life of 15.5 years, while 6-MM had only 57% 10-year survival with graft half-life of 11.8 years (**Figures 3A, B**; **Table 3**). The same pattern was for primary/re-transplant or just primary (not shown) transplants, as was previously described (1, 2).

**Table 3:** Graft half-lives based on death-censored graft survival analyses using the different immunogenicity scales in the primary/re-transplants cohort. Immunogenicity was calculated based on HLA-A/B/DR loci. Graft half-life values over 16.1 years were imputed as described in *Materials and Methods* section.

Immunogenicity Cutoff value	Graft Half-Life, years (95% CI*)		n	
	Below Cutoff	Above Cutoff	Below Cutoff	Above Cutoff
0-6 MM scale**				
Antigenic Match (MM = 0)	15.5 (15.1–NA)	13.4 (13.1–13.7)	5,971	72,208
Predictive Threshold (MM = 3)	15.1 (14.6–15.6)	13.0 (12.7–13.3)	19,826	58,353
Weakly Immunogenic (MM ≤ 1)	16.1 (15.1–NA)	13.3 (13.0–13.6)	6,723	71,456
Median (HLA MM = 4)	14.4 (14.1–14.9)	12.9 (12.3–13.2)	39,853	38,326
**HLA-A/B/DR HMS scale**				
Allelic Match (HMS = 0)	18.1 (15.1–NA)	13.4 (13.1–13.7)	4,899	73,884
Predictive Threshold (HMS = 1.8)	17.0 (15.1–NA)	13.3 (13.3–13.0)	7,846	70,945
Weakly Immunogenic (HMS = 3.0)	16.7 (15.7–NA)	12.9 (9.8–13.8)	11,483	67,381
Median (HMS ≤ 7.8)	14.3 (14.0–14.6)	12.5 (12.2–13.0)	39,439	39,425
**HLA-A/B/DR EMS scale**				
Allelic Match (EMS = 0)	17.1 (15.1–NA)	13.4 (13.1–13.7)	4,701	74,163
Predictive Threshold (EMS = 1.8)	16.2 (15.1–NA)	12.8 (12.4–13.2)	7,858	71,006
Weakly Immunogenic (EMS = 3.6)	16.7 (15.1–NA)	13.2 (12.9–13.6)	14,165	64,699
Median (EMS = 7.9)	14.4 (14.2–15.0)	12.8 (12.4–13.1)	39,226	39,638
**HLA-A/B/DR AMS scale**				
Allelic Match (AMS = 0)	17.1 (15.1–NA)	13.4 (13.1–13.7)	4,899	73,965
Predictive Threshold (AMS = 2.5)	15.6 (15.1–NA)	13.2 (12.9–13.5)	9,782	69,082
Weakly Immunogenic (AMS = 2.3)	15.3 (15.0–NA)	13.1 (12.9–13.4)	9,712	69,647
Median (AMS = 5.8)	14.4 (14.1–14.8)	12.9 (12.4–13.2)	25,841	53,023
**HLA-A/B/DR Antibody-Verified Eplet load scale**				
Allelic Match (EpMM = 0)	22.6 (NA–NA)	15.3 (15.0–15.6)	1,584	45,657
Predictive Threshold (EpMM = 2)	17.5 (NA–NA)	15.1 (14.9–15.6)	6,335	40,906
Weakly Immunogenic (EpMM = 1.7)	18.3 (NA–NA)	15.1 (14.9–15.6)	5,419	41,882
Median (EpMM = 5.5)	15.2 (14.4–16.1)	13.5 (13.2–13.8)	21,170	20,071

*NA in confidence intervals indicates the upper survival limit was too far in the future to be computed in PROC LIFETEST procedure.

**The total numbers of patients in HLA MM categories is 78,179, reflecting records with blank HLA MM variable values.

Our goal was to select an HMS threshold with the largest number of patients producing graft survivals as in a 0-/1-HLA-A/B/DR MM cohort. In particular, we stratified transplant recipients into four immunological risk categories based on HMS (EMS, AMS, or EpMM) thresholds and analyzed their graft failure rates in Kaplan–Meier survival estimates. The HMS-based thresholds were HMS=0 (perfect allelic match), HMS=1.8 (predictive threshold), HMS=3.0 (low IM threshold; equal to mean HMS in 0-/1-HLA MM), and HMS=7.8 (median HMS threshold). EMS, AMS, and EpMM-based thresholds were defined similarly and their corresponding values (**Table 3**).

**Figure 4:**
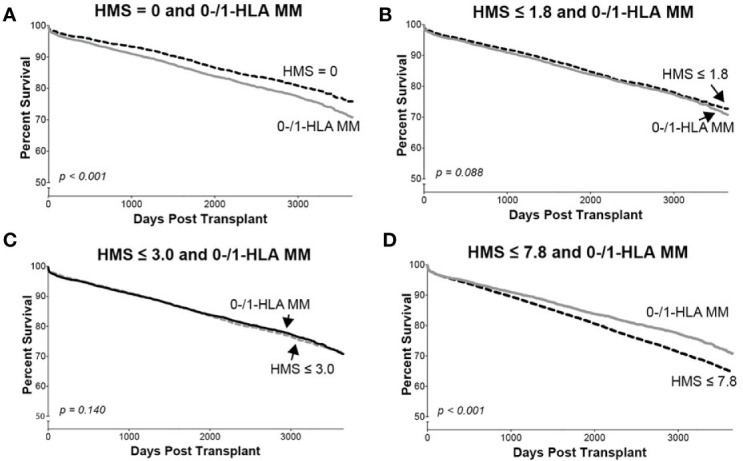
The Kaplan–Meier death-censored graft survival in patients selected by different HMS thresholds compared to patients with combined 0-/1-HLA MM selected from the primary/re-transplant cohort (n = 78,865). Patients with HMS=0 **(A)**, patients with HMS≤ 1.8 **(B)**, patients with HMS ≤ 3.0 **(C)**, and patients with HMS≤ 7.8 **(D)** were compared to the patients in the same cohort with 0-/1-HLA MM.

In detailed analysis, the perfect allelic match HMS=0 selected 4,899 patients with graft half-life of 18.1 years, gaining 5.0 years over 13.1 years for the HMS>0 group (p < 0.001; **Figure 3C**; **Table 3**). The predictive threshold based on the maximally-selected statistics (29) calculated an HMS=1.8 as low-risk predictor for the graft loss (p < 0.001; **Figure 3D**). The HMS ≤ 1.8 group had 7,846 patients with 16.6-year graft half-life, a 3.6-year improvement over 13.0 years in the HMS>1.8 group (p > 0.001; **Figure 3D**; **Table 3**). In another analysis, with the 0–10 HMS integer scale, the most gain for the graft survival was observed in transplants with HMS up to 3.0. This HMS ≤ 3.0 threshold included 11,483 transplants with 16.7-year graft survivals, a 3.8-year gain over 12.9-year in the HMS>3.0 group (p < 0.001; **Figure 3E**; **Table 3**). Finally, the median HMS ≤ 7.8 cohort achieved 14.3-year survival, a 1.8-year better than 12.5 years for HMS>7.8 cohort (p < 0.001; **Figure 3F**; **Table 3**). Notably, the transplants with HMS ≤ 1.8 and HMS ≤ 3.0 had graft survival better or equal to that in the 0-/1-HLA MM cohort (**Figure 4**); however, the number of patients in HMS ≤ 1.8 and HMS ≤ 3.0 was significantly higher than in the 0-/1-HLA MM cohort (**Table 3**). The EMS, AMS, and EpMM values showed similar patterns with their own specific thresholds; EpMM evaluation displayed comparable results (**Table 3**).

### The Dominant Role of -DR With -DQ HMS Values in Kidney Allograft Survival

**Figure 5:**
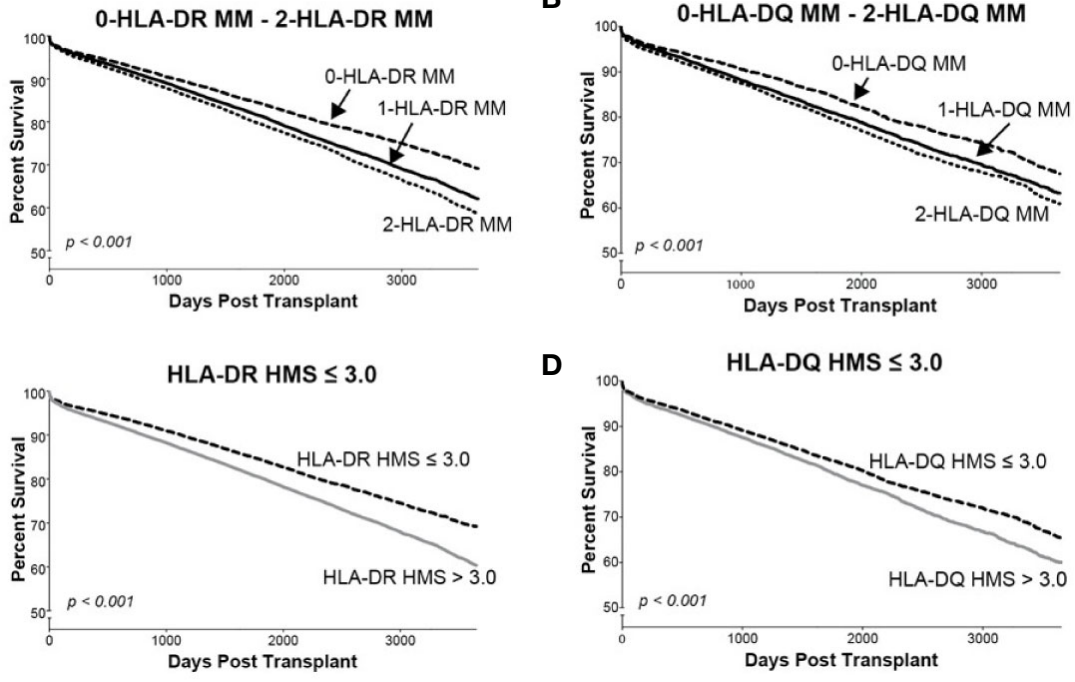
The Kaplan–Meier death-censored graft survival as a function of class II (HLA-DR and –DQ) immunogenicity in primary/re-transplant cohorts (n = 78,865). Panels **(A, B)** present patients stratified by mismatches at 0-, 1- or 2-HLA-DR **(A)** or -DQ **(B)** antigens. Transplants from donors with split broad antigens (for example, HLA-DR3 in donor and HLA-DR17 in recipient—this could be either a match or a mismatch) were excluded from the analysis; Panel **(C)** presents patients stratified by HLA-DR HMS ≤ 3.0 *vs*. HLA-DR HMS>3.0, and Panel **(D)** presents patients stratified by HLA-DQ HMS ≤ 3.0 *vs*. HLA-DQ HMS>3.0.

Since donor class II -DR and -DQ mismatches proved to be very important in kidney allograft survivals (19, 34–36), we examined their impact at the same HMS thresholds. The 29,852 HLA-A/B/DR/DQ cohort showed that recipients matched at -DR alone had grafts surviving 15.3 years while those matched at -DQ alone 15.0 years (**Table 4**). The HMS=0 group matched at HLA-A/B/DR exhibited an extra benefit from an additional matching at -DQ with 18.4 years *vs*. 15.6-year survivals with mismatch at -DQ, an almost 3.0-year benefit (**Table 4**); similar gains were produced by -DQ matching at HMS ≤ 3.0 and HMS ≤ 7.8 (**Table 4**). The importance of -DR over -DQ was shown when the entire cohort was matched *vs*. mismatched at only -DR (**Figure 5A**) as well as matched *vs*. mismatched at only -DQ (**Figure 5B**), as the benefit of additional -DQ match in -DR matched transplants was not statistically significant. Similar patterns were also displayed when HMS ≤ 3.0 was calculated only for -DR or -DQ (**Figures 5C, D**). However, patients with HMS matched at HLA-A/B/DR had an extra benefit from an additional -DQ match (**Table 4**, **Supplemental Figures 1A–C**). This benefit was stronger from -DR matching in transplants already matched by -DQ than *vice versa* (**Supplemental Figures 1D, E**). Our conclusion is that the -DRB1 locus plays the dominant role in the HMS values, but additional matching at -DQB1 locus improves an overall graft survival.

**Table 4:** HLA-class II immunogenicity and graft survival. Graft half-lives based on death-censored graft survival analyses with 0- to 2-HLA MM scale and the flexible HMS scale in the primary/re-transplants cohort. Immunogenicity was calculated based on HLA- DRB1 and/or HLA-DQB1 loci.

HLA-DRB1 or -DQB1	N	Graft half-life, years (95% CI)	95% CI*
HLA-DR MM = 0	15,014	15.3	15.1–NA
HLA-DQ MM = 0	5,775	15.0	14.3–NA
**HLA-A/B/DR/DQ**	**N**	**Graft half-life, years (95% CI)**	95% CI*
A/B/DR HMS = 0, -DQ MM = 0	1,864	19.2	NA–NA
A/B/DR HMS = 0, -DQ MM = 1 or 2	1,068	18.1	15.1–NA
A/B/DR HMS ≤ 3.0, -DQ MM = 0	2,773	18.4	15.0–NA
A/B/DR HMS ≤ 3.0, -DQ MM = 1 or 2	2,846	15.6	15.1–NA
A/B/DR HMS ≤ 7.8, -DQ MM = 0	4,538	15.3	14.6–NA
A/B/DR HMS ≤ 7.8, -DQ MM = 1 or 2	11,289	13.9	13.1–15.1
**Matched by HLA-DRB1**	**N**	**Graft half-life, years (95% CI)**	95% CI*
HLA-DR MM = 0, HLA-DQ MM = 0	3,883	15.3	15.0–NA
HLA-DR MM = 0, HLA-DQ MM = 2	624	16.1	9.7–NA
**Matched by HLA-DQB1**	**n**	**Graft half-life, years (95% CI)**	95% CI*
HLA-DQ MM = 0, HLA-DR MM = 0	3,883	15.3	15.0–NA
HLA-DQ MM = 0, HLA-DR MM = 2	457	12.7	13.9–NA

*NA in confidence intervals indicates the upper survival limit was too far in the future to be computed in PROC LIFETEST procedure.

### Simulated Matching to Generate the Maximum Possible Number of Weakly-Immunogenic Donor/Recipient Pairs

So far, only 6% of deceased donor transplants were fully matched (11), while 83.6% of transplants had more than three HLA-A/B/DR mismatches. The new kidney allocation policy by OPTN (Organ Procurement and Transplantation Network) establishes a 250-mile radius procurement regions for deceased kidney harvesting, assigning in the order of ≈1,000 deceased kidneys per region (37). We tested whether HMS thresholds could reshuffle donors to recipients to maximize those with HMS ≤ 3.0, HMS ≤ 5.5, or HMS ≤ 7.8 (**Table 5**). Random donor/recipient pair populations were re-matched to form the maximum possible number of new pairs that would have HMS or HLA MM value within the designated IM threshold. ABO compatibility was or was not enforced in simulated pairs. This approach re-matched up to 85% of pairs under HMS ≤ 3.0 in the population of 5,000 input pairs (**Table 5**). Furthermore, enforcing HMS ≤ 7.8 threshold produced 95% matches for 1,000 and 99% for 5,000 random pairs. Re-matched weakly immunogenic donor/recipient pairs had recipient ABO distribution similar to that in the general population (**Supplemental Table 3**). Enforcing ABO matching produced slightly lower percentages of donor/recipient pairs that could be re-matched, though that difference was small in n = 1,000 and n = 5,000 trial sizes (**Table 5**). Remarkably, re-matching of 1,000 pairs to obtain the largest number of 0-/1-HLA MM pairs produced only 12.5% ( ± 0.85%) matches (**Table 5**). Thus, a continuous IM scale may find low IM donors for most patients and may exclude all transplants with high IM donors (HMS>7.8).

**Table 5:** Simulation analyses to re-match 10, 100, 1,000 or 5,000 donor/recipeint pairs with HMS = 0, HMS ≤ 3.0, ≤ 5.5, or ≤ 7.8.

Immunogenicity threshold	ABO-matched	Percent of re-matched pairs			
		n* = 10	n = 100	n = 1,000	n = 5,000
**HMS = 0**	No	1.00	6.90	16.10	23.53
	Yes	2.00	6.30	14.01	22.05
**HMS ≤ 3.0**	No	15.91	44.00	72.98	84.85
	Yes	7.00	32.60	63.61	79.07
**HMS ≤ 5.5**	No	47.00	71.40	88.00	93.20
	Yes	26.00	60.70	83.10	89.96
**HMS ≤ 7.8**	No	58.00	81.60	95.33	98.72
	Yes	46.00	73.60	90.32	96.26
**HLA MM ≤ 1**	No	1.00	3.20	14.49	ND**

Simulation program removes all donor-recipient pairs that are ABO-incompatible or have HMS greater than the indicated thresholds and rearranges the pairs to achieve the maximum number of pairs with HMS = 0, ≥ 3.0, ≥ 5.5, or ≥ 7.8, or HLA MM ≤ 1. The experiment with 1,000 pairs was repeated 10 times, whereas the experiment with 5,000 pairs was repeated once.

*n – population of donor/recipients pair size.

**ND – not done.

## Discussion

The main message is that a continuous HLA IM scale finds low-IM donors better than the current HLA mismatch scale. We report herein four main findings. First, the IM, calculated for HLA-A/B/DR/DQ of each donor/recipient pair by the Cambridge algorithm (HMS) correlated with long-term kidney allograft survivals. Second, low IM donors were selected on a flexible scale ranging from zero to median HMS values. Third, the HMS ≤ 3.0 threshold had an excellent 16.7-year graft survival with a 3.8-year benefit over HMS>3.0 transplants; the median HMS ≤ 7.8 threshold had 14.2-year graft survival, a 2.8-year benefit. Fourth, 75% of 1,000 random ABO-matched patients were then re-matched with HMS ≤ 3.0 value and the remaining 25% with the medium HMS 3.0–7.8 value. Overall, such approach in our cohort would increase the graft half-life from 13.5-years to 16.4 years. Thus, the high-resolution HLA typing with the HMS-based matching could revolutionize the selection of donors.

The 2-digit 0-HLA MM group with graft survival of 15.5 years included the “real” 4-digit 0-HLA MM transplants (HMS = 0) with superior graft survival of 18.1 years. Thus, only precise 4-digit HLA matching produced “perfect” matches with superior outcomes, otherwise achieved only by living related donors (38). This was as equally impressive as 16.7-year graft half-life among 11,483 patients with HMS ≤ 3.0 selected from the 0- to 6-MM cohort (**Table 3**). The median HMS ≤ 7.8 threshold had graft survivals of 14.3 years and that should be the upper limit to avoid high IM transplants (HMS > 7.8). The survival benefit in weakly immunogenic transplants is excellent in recipients of kidneys with full allelic match, but when HMS=0 patients were excluded from HMS ≤ 3.0 cohort, the graft survival still was significantly higher than in HMS>3.0 cohort (14.6 *vs* 12.9 years, not shown). While HLA-DR was dominant in contributing to the HMS benefit, matching at -DQ locus further improved allograft survival in patients with low HMS scores (**Table 4**); the -DQ analysis was limited by 30% availability of this locus typing in the SRTR database. Overall, we propose that the Cambridge algorithm proffers a versatile way to identify low/medium IM donors independent of 0-6 HLA matching: the best results were produced by HMS matching at the combined HLA-A/B/DR/DQ loci.

A continuous IM scale is versatile. In the retrospective cohort of 78,864 recipients, there were 11% more pairs with HMS ≤ 1.8 than those with 0-/1-HLA MM. Similarly, there were 35% more pairs with HMS ≤ 3.0 than those with 0-/1-HLA MM. Given the median graft survival in HMS ≤ 1.8 and HMS ≤ 3.0 groups was comparable to that in the 0-/1-HLA MM group (**Table 3**), it is easy to see that substantially better survival results could be produced if more effort was put in finding weakly immunogenic donors on a continuous IM scale. Exploring this insight, our simulation found that 75% of 1,000 random pairs could be re-matched with low (HMS ≤ 3.0) and remaining 25% with medium (HMS 3.0–7.8) IM donors (**Table 5**). This would increase an average graft survival by 21% from 13.5 years to 16.4 years producing extra 224,762 kidney life years in 78,864 patients. While we did take into consideration ABO compatibility, unacceptable HLA antigens data was not available in the SRTR database. Still, HMS alone and ABO blood groups/HMS re-matching simulation provided a proof of concept: a random 10 to 5,000 pool of donor/recipient contain sufficiently diverse HLA haplotypes to accommodate most if not all recipients with weakly immunogenic grafts. We plan to quantify benefits using real-life simulation conditions.

The proportional hazard regression already examined the impact of HLA on graft survival with multiple covariates (2). This multifactorial analysis provided tools for addressing assumptions violations in the Cox model. Proportional hazard distribution is one of the most important prerequisites in a model validation (39, 40). Williams and his colleagues did not find substantial deviations from the proportionality in their research cohorts (2). Our data demonstrated significant non-proportionality with most confounders, prompting our use of time log transformation to rectify our model (33). It is possible that the strict definition of proportionality could have contributed to the difference. Despite this disparity, both Cox analyses revealed that immunosuppression and induction therapy had the highest impact (p < 0.001) on the IM-dependent graft survival (2). Thus, immunosuppression and induction covariates are affecting the HLA-based matching of donors and recipients.

While imputation is not recommended for clinical decision making (39), it is an invaluable tool for research. We have developed an algorithm for fast imputation of high-resolution HLA types on the HaploStats server (26). The proportion of HLA alleles imputed with high likelihood was shown on 5,000 volunteers with 91% accuracy for HLA-A, 88% for -B, 67% for -DRB1, and 95% for -DQB1 (an average of 85%) in previously published research (27). Our analysis of 1,095 individuals produced better results: the imputed/real high-resolution was 96% precise for HLA-A locus, 92% for -B locus, 73% for -DRB1 locus, and 85% for -DQB1 locus (**Table 2**). Future clinical application needs *bona fide* 4-digit HLA typing rather than imputed HLA identification.

The half-life of kidney grafts from deceased donors has slightly improved from 7.4 years in 1994 to 9.8 years in 2008 (11). An independent 20-year study confirmed graft half-life of 9.3 years in 2015 (41). The 2-year improvement can be attributed to better induction and immunosuppression therapy in almost all recipients (42, 43). In the U.S., HLA matching is not done because of the logistic complexity of the 0- to 6-HLA scale and the desire to avoid disadvantaging minority patients. The sensitization of patients further complicates selection of donors (44–46). Our approach may help avoid unacceptable HLA alleles when finding low IM donors.

Analysis of donor eplets, based on 4-digit HLAs, became necessary to overcome an increasing number of new HLA alleles (47). In fact, one very immunogenic eplet may be present on multiple HLA molecules requiring their avoidance in donor selection. The low HLA eplet mismatch (EpMM) load has been considered as an extension of 2-digit HLA antigens in improving donor choice, especially for children (48). Indeed, modified Poisson regression showed that the EpMM load was an effective tool in the risk assessment. In other work, EpMM values were correlated with AMS and EMS scores (R^2^ = 0.85–0.96) in *de novo* donor-specific antibody (dnDSA) production (19). Furthermore, all three methods (EpMM, EMS, and AMS) correlated with dnDSA following adjustment of variables such as recipient age, immunosuppression, and non-compliance. Overall, the authors suggested to use the easiest method as similar results were produced by each method. We argue about the use of HMS, EMS or AMS values. However, we also recognize the qualitative differences among antibody-verified eplets as more or less immunogenic, as others showed (47, 49, 50). Thus, we believe that future approach should combine the HMS threshold with antibody-verified EpMM analysis for the best pre-transplant risk assessment.

In summary, we propose to prioritize low IM donors by utilizing HMS-based selection instead of the rigid 0–6-HLA system. Relying on physicochemical properties provides flexibility and may accommodate all or almost all recipients. The application of the HMS system for all deceased donor kidney transplants in United States should significantly increase graft survivals.

## Data Availability Statement

Publicly available datasets were analyzed in this study. The datasets analyzed for this study can be obtained upon request from the Scientific Registry of Transplant Recipients (SRTR) [www.srtr.org].

## Author Contributions

DB, BM, MR and SS guided the research and wrote the manuscript. RG performed high-resolution HLA types imputation and re-matching simulation analyses. JBo and SK consulted and performed the statistical analyses. RL and AG performed 4-digit and 2-digit HLA identification in 1,095 patients, their statistical evaluation and HLA typing data analyses. BG, SL-M and JBr provided intellectual support for data processing and analyses. All authors contributed to the article and approved the submitted version.

## Funding

Research reported in this publication was supported by the National Library of Medicine of the National Institutes of Health under Award Number R01LM013311 as part of the NSF/NLM Generalizable Data Science Methods for Biomedical Research Program. The content is solely the responsibility of the authors and does not necessarily represent the official views of the National Institutes of Health. Additional funding was provided by The Alliance for Paired Donation, Maumee, OH, USA.

## Conflict of Interest

JB was employed by Parexel International.

The authors declare that the research was conducted in the absence of any commercial or financial relationships that could be construed as a potential conflict of interest.
