# Supplementary material for: Low Hydrophobic Mismatch Scores Calculated for HLA-A/B/DR/DQ Loci Improve Kidney Allograft Survival
Source: Front Immunol. 2020 Oct 29;11:580752. doi: 10.3389/fimmu.2020.580752 (PMC7659444; doi:10.3389/fimmu.2020.580752)
Supplement: Supplementary file 1 [file Table_1.docx]

Supplementary Material

##
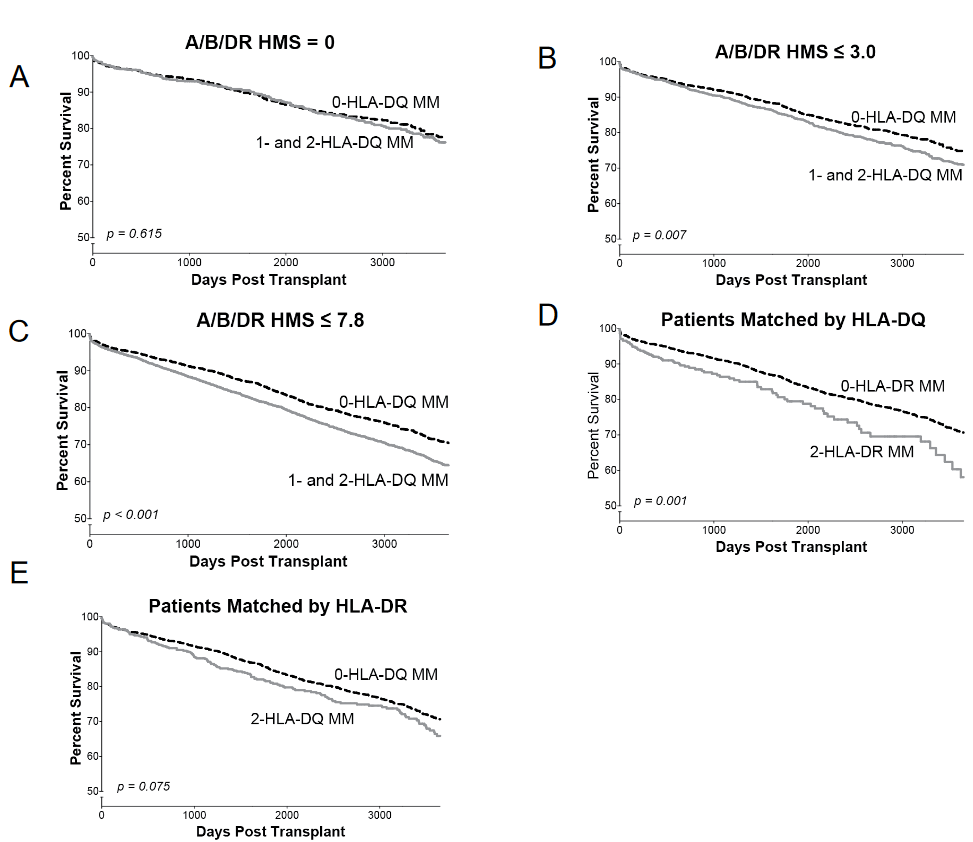
Supplementary Figures

## Supplemental Figure 1.

## The death-censored graft survival in primary/re-transplant cohort matched by HLA-A/B/DR HMS, with A/B/DR HMS=0 (A), A/B/DR HMS≤3.0 (B) or A/B/DR HMS≤7.8 (C) and stratified by HLA-DQ antigenic mismatch (DQ MM=0 or >0). Panels D and E show the death-censored graft survival in primary/re-transplant cohort (n=78,865). Panel D: patients matched by HLA-DR (-DR MM=0) and stratified by antigenic HLA-DQ mismatch (-DQ MM=0 or -DQ MM=2). Panel E: patients matched by HLA-DQ (-DQ MM=0) and stratified by antigenic HLA-DR mismatch (-DR MM=0 or -DR MM=2).

## Supplementary Tables

**Supplemental Table 1.** Confounders for Cox regression.

| **Variable** | **Reference category*** | **Category that is compared to the reference** | **Hazard ratio (p-value) in univariate regression** |
| --- | --- | --- | --- |
| **Donor age** | 18-34 years old | 35-49 years old | 1.327 (<0.0001) |
| **Recipient age** | 18-34 years old | 35-49 years old | 0.673 (<0.0001) |
| **Donor gender** | M | F | 1.145 (<0.0001) |
| **Peak PRA** (continuous) | NA | NA | 1.003 (<0.0001) |
| **Transplant era** | 2000-2004 | 2005-2016 | 0.781 (<0.0001) |
| **Induction therapy** | No | Yes | 0.817 (<0.0001) |
| **Maintenance immunosuppression**** | TAC/MMF | TAC | 1.257 (<0.0001) |
| **Recipient race** | Caucasian | African American | 1.639 (<0.0001) |
| **Donor race** | Caucasian | African American | 1.324 (<0.0001) |
| **Recipient education** | Up to high school | College or higher | 0.906 (<0.0001) |
| **Recipient primary source of payment** | Private insurance | Public insurance | 1.158 (<0.0001) |
| **Cold ischemia time** | 24h or less | Over 24h | 1.188 (<0.0001) |
| **Waiting time** | Less than 1 year | 3 to 5 years | 1.106 (0.0001) |
| **Candidate BMI** | Less than 30 | Over 30 | 1.123 (<0.0001) |
| **Candidate pre-transplant diabetes** | No diabetes | T1D | 0.880 (0.0156) |
| **Pre-transplant dialysis** | No | Hemodialysis | 1.569 (<0.0001) |
| **HLA-A/B/DR mismatch** | 0 | 2 | 1.189 (0.0005) |
| **Deciles of HMS scores** | First | Second | 1.372 (<0.0001) |

* The hazard ratios and p-values are reported in comparison to reference categories. For example, apparently from row 1 of this table, recipients of transplants with donor age 35-49 years had a hazard ratio of graft failure of 1.327 compared to transplants with donor age 18-34 years.

** Both TAC/MMF and TAC regimen values for maintenance immunosuppression may or may not include steroids.

**Supplemental Table 2.** Multiple Cox regression analysis of the primary/re-transplant recipients cohort.

| **Variable**  **(ref vs comparison)** | **Reference category** | **Category that is compared to the reference** | **Hazard ratio** | **p-value** |
| --- | --- | --- | --- | --- |
| Donor age | 18 to 34 years | 35 to 49 years | 1.417 | 0.0028 |
| Recipient age | 18 to 34 years | 35 to 49 years | 0.796 | 0.0689 |
| Donor gender | Male | Female | 1.230 | 0.0099 |
| Peak PRA | NA | NA | 1.002 | <0.0001 |
| Transplant era | 2000-2004 | 2005-2016 | 1.277 | 0.0275 |
| Induction | No | Yes | 1.598 | <0.0001 |
| Maintenance immunosuppression | TAC/MMF/PRED | TAC/PRED | 2.394 | <0.0001 |
| Recipient race | Caucasian | African American | 0.828 | 0.0399 |
| Donor race | Caucasian | African American | 1.368 | 0.0025 |
| Recipient education | None, grade school or high school | Attended college, bachelors or post-college | 1.184 | 0.0389 |
| Recipient primary source of payment | Private insurance | Public insurance | 1.225 | 0.0445 |
| Cold ischemia time | Less than 24h | Greater than 24h | 1.657 | <0.0001 |
| Waiting time | Less than 1 year | 3 to 5 years | 0.946 | 0.0685 |
| Candidate BMI > 30 | No | Yes | 1.203 | 0.0272 |
| Candidate pre-transplant diabetes | No | Type I diabetes | 1.034 | 0.6232 |
| Pre-transplant dialysis | No | Hemodialysis | 1.377 | 0.0063 |
| HMS deciles | 1^st^ decile | 2^nd^ decile | 1.241 | <0.0001 |

Akaike Information Criterion =168,165.92**Supplemental Table 3.** Distribution of recipient ABO types in pairs re-matched in allocation simulation analysis. In simulation, re-matching was performed in 100, 1,000, or 5,000 donor/recipient pair populations such that maximum number of new pairs had HMS=0, HMS≥3.0, ≥5.5, or ≥7.8. The recipient ABO types in the new low IM pairs are reported under “% Re-matched recipients by ABO”.

| **Population size** |  | **HMS cutoff** | | | |
| --- | --- | --- | --- | --- | --- |
|  |  | **HMS = 0** | **HMS≥3.0** | **HMS≥5.5** | **HMS≥7.8** |
| 100 | **% Re-matched** | 6.30 | 32.60 | 60.70 | 73.60 |
|  | **% of Re-matched recipients by ABO** | | | | |
|  | A | 40.00% | 36.00% | 47.00% | 29.00% |
|  | B | 10.00% | 10.00% | 11.00% | 18.00% |
|  | AB | 4.00% | 8.00% | 7.00% | 7.00% |
|  | O | 46.00% | 46.00% | 35.00% | 46.00% |
| 1,000 | **% Re-matched** | 14.01 | 63.61 | 83.10 | 90.32 |
|  | **% of Re-matched recipients by ABO** | | | | |
|  | A | 38.80% | 37.00% | 36.80% | 37.2% |
|  | B | 13.30% | 13.20% | 14.60% | 15.90% |
|  | AB | 6.30% | 5.50% | 5.20% | 6.90% |
|  | O | 41.60% | 44.30% | 43.40% | 40.00% |
| 5,000 | **% Re-matched** | 22.05 | 79.07 | 89.96 | 96.26 |
|  | **% of Re-matched recipients by ABO** | | | | |
|  | A | 38.26% | 37.10% | 38.20% | 36.64% |
|  | B | 12.98% | 13.88% | 13.44% | 14.76% |
|  | AB | 5.26% | 5.72% | 5.30% | 6.08% |
|  | O | 43.50% | 43.30% | 43.06% | 42.52% |
